# Direct Vibrational Stark Shift Probe of Quasi-Fermi Level Alignment in Metal Nanoparticle Catalyst-Based Metal–Insulator–Semiconductor Junction Photoelectrodes

**DOI:** 10.1021/jacs.3c02333

**Published:** 2023-06-22

**Authors:** Sa Suo, Colton Sheehan, Fengyi Zhao, Langqiu Xiao, Zihao Xu, Jinhui Meng, Thomas E. Mallouk, Tianquan Lian

**Affiliations:** † Department of Chemistry, Emory University, Atlanta, Georgia 30322, United States; ‡ Department of Chemistry, University of Pennsylvania, Philadelphia, Pennsylvania 19104, United States

## Abstract

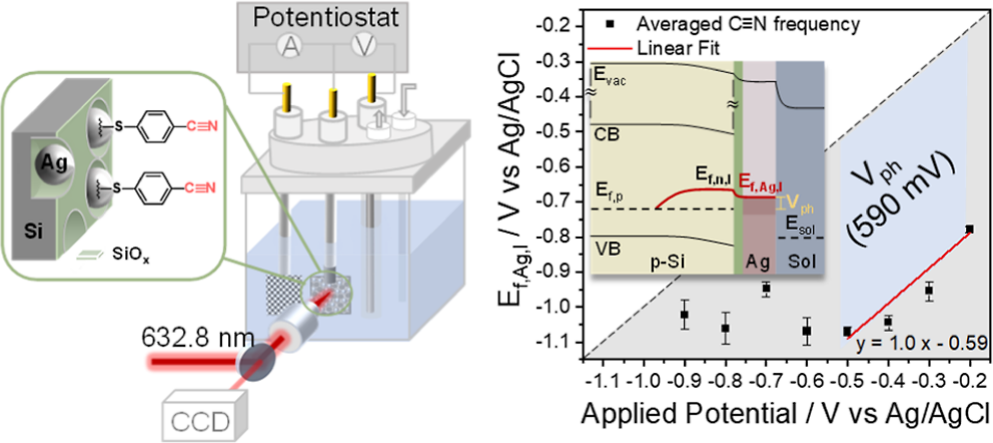

Photoelectrodes consisting of metal–insulator–semiconductor (MIS) junctions are a promising candidate architecture for water splitting and for the CO_2_ reduction reaction (CO_2_RR). The photovoltage is an essential indicator of the driving force that a photoelectrode can provide for surface catalytic reactions. However, for MIS photoelectrodes that contain metal nanoparticles, direct photovoltage measurements at the metal sites under operational conditions remain challenging. Herein, we report a new in situ spectroscopic approach to probe the quasi-Fermi level of metal catalyst sites in heterogeneous MIS photoelectrodes via surface-enhanced Raman spectroscopy. Using a CO_2_RR photocathode, nanoporous p-type Si modified with Ag nanoparticles, as a prototype, we demonstrate a selective probe of the photovoltage of ∼0.59 V generated at the Si/SiO_*x*_/Ag junctions. Because it can directly probe the photovoltage of MIS heterogeneous junctions, this vibrational Stark probing approach paves the way for the thermodynamic evaluation of MIS photoelectrodes with varied architectural designs.

## Introduction

The development of sustainable and low-cost solar energy conversion technologies is one of the most important scientific challenges today. Photoelectrochemical (PEC) cells are a promising technology for converting solar energy into clean chemical fuels by producing H_2_ via water splitting,^1^ carbon-based fuels via CO_2_ reduction reactions,^2^ and NH_3_ via nitrogen reduction.^3^ The key components of PEC cells are the photoanode and photocathode, on which light-driven oxidation and reduction reactions occur, respectively. Their properties determine the overall performance of PEC cells. A cost-effective and versatile design of photoelectrodes is based on metal–insulator–semiconductor (MIS) junctions, which consist of a catalytic metal layer that forms a Schottky contact with the semiconductor and an insulator layer that serves as a protecting and charge tunneling layer on top of the semiconducting light-absorbing substrate (see below).^4,5^ One of the key parameters for high-performing MIS photoelectrodes is their ability to generate a photovoltage, that is the splitting between the quasi-Fermi levels of the minority and majority carriers because it provides the thermodynamic driving force for catalytic reactions on the metal surface. This quantity depends sensitively on the properties of the semiconductor, insulating layer (e.g., charge extraction barrier,^6^ thickness,^7−10^ and surface passivation ability^11−14^), and the work function of the metal.^15,16^ The rational design and improvement of such MIS-based photoelectrodes require in situ methods of measuring the photovoltage generated at these heterogeneous junctions.

The traditional PEC method for estimating the photovoltage of a photocathode, taking a p-type semiconductor as an example, is to measure the potential difference between the photocurrent in the p-type photocathode under illumination and the dark current of the degenerately doped n-type cathode at the same current density.^17,18^ This method is based on the assumption that both electrodes have an identical metal surface structure, catalytic reaction rate, insulator thickness, and insulator/metal contact, which can be difficult to ensure in many cases. Furthermore, for electrodes with metal nanoparticles, the measured potential difference is a composite photovoltage of the electrolyte–insulator–semiconductor and electrolyte–MIS junctions. Kelvin probe force microscopy can achieve nanometer scale imaging of the surface photovoltage, but this technique cannot be applied under operando PEC conditions.^19^ More recently, a direct in situ electrical measurement of the potential response of the catalyst layer at semiconductor/catalyst junctions via the dual-working-electrode (DWE) PEC technique was reported.^20^ This technique enables the independent modulation and direct probing of the Fermi level at the bulk semiconductor substrate through the first working electrode and the quasi-Fermi level at the catalytic layer via the second working electrode (an ion-permeable Au contact at the catalyst surface).^20,21^ However, the DWE technique is not applicable to nanoscale heterogeneous interfaces. Complementarily, the potential-sensing electrochemical atomic force microscopy (PS-EC-AFM) technique is capable of spatially resolving the operando photovoltage at each semiconductor/catalyst junction.^22^ This technique requires intimate and reproducible contact between the AFM tip and the target layer, which can be difficult to achieve in some cases. Thus, there is a need for alternative techniques for contactless direct probing of the quasi-Fermi level at metal particle catalysts on semiconductor photoelectrodes.

Here, we report for the first time an in situ spectroscopic method for directly probing the quasi-Fermi level of metal particle catalysts on semiconductor photoelectrodes by surface-enhanced Raman spectroscopy (SERS). We demonstrate this method on a MIS-based CO_2_ reduction photocathode consisting of p-type black silicon (p-b-Si), a native oxide layer, and silver nanoparticles.^23^ Using 4-mercaptobenzonitrile (4-MBN), a vibrational Stark effect probe molecule that can selectively adsorb on Ag nanoparticles, we show that its vibrational frequency shifts with the majority carrier Fermi level of p-Si. By comparison with the potential-dependent frequencies on Ag electrodes, we can determine the quasi-Fermi level of the Ag nanoparticle on p-Si, revealing a photovoltage of ∼590 mV for these electrodes. The direct probe of the quasi-Fermi level in the metal catalyst enables a more accurate estimation of the available photovoltage generated at MIS photoelectrodes, making this a useful tool for in situ probing of photovoltages on MIS junctions.

## Results

**Figure 1:**
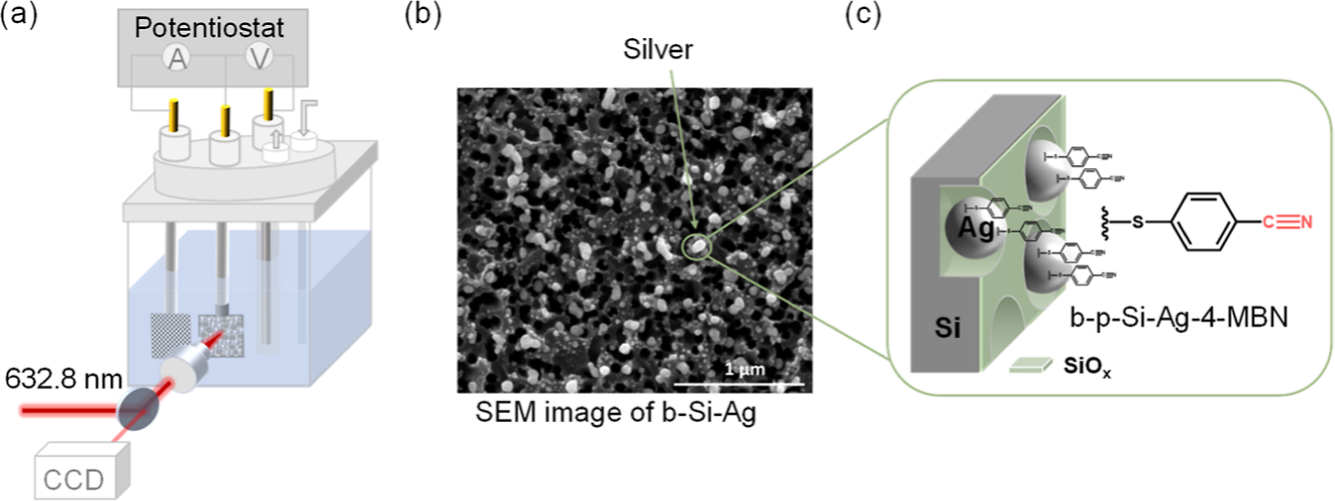
(a) Schematic illustration of the three-electrode (photo)electrochemical setup. The three electrodes from left to right are the counter electrode (Pt mesh), working electrodes (b-p-Si–Ag NPs–4-MBN or roughened Ag–4-MBN), and reference electrode (Ag/AgCl/1 M KCl). The solution is 0.2 M KHCO_3_ (pH = 8.2). The 632.8 nm Raman laser beam is focused onto the working electrode surface and the Raman scattered light is collected by an electron-multiplied charge coupled device (see Supporting Information, Section 3 for a detailed description of the home-built Raman setup). (b) SEM image of the p-type black silicon–Ag composite. (c) Schematic illustration of the photoelectrode structure: b-p-Si–Ag NPs with 4-mercaptobenzonitrile (4-MBN) selectively bonded to Ag via its thiol anchoring group.

The in situ SERS and electrochemical measurements were conducted using a CHI660e workstation (CH Instruments) in a three-electrode setup, shown in Figures 1a, S1, and S2. The working electrode is roughened Ag, or a black p-doped Si electrode functionalized with Ag nanoparticles (b-p-Si–Ag), the reference electrode is Ag/AgCl (in 1 M KCl), and the counter electrode is a Pt mesh. The electrolyte is 0.2 M KHCO_3_ solution (pH = 8.2). All potentials referred to in the paper are relative to Ag/AgCl/1 M KCl. The details for preparing the working electrodes are described in the Supporting Information (Sections 1.1 and 1.2).

Figure 1b shows a representative SEM image of the b-p-Si–Ag photoelectrode. It consists of a p-type nanoporous silicon photoelectrode with an average pore diameter of ∼80 nm, a native SiO_*x*_ layer with an estimated thickness of a few nanometers and silver nanoparticles (∼50–100 nm in diameter) on the electrode surface. This electrode structure has been shown to lead to a more anodic photocurrent onset potential and enhanced selectivity for CO_2_ reduction to CO compared to p-type nanoporous silicon photoelectrodes (without silver nanoparticles).^23^ To enable the vibrational Stark effect measurement of the Fermi level at the Ag particle, 4-MBN, which selectively binds to the Ag surface via its thiol linking group, was used as the Stark shift probe molecule.^24−26^ 4-MBN molecules were adsorbed onto the electrode surface, following the procedure described in Supporting Information, Section 1.3.

**Figure 2:**
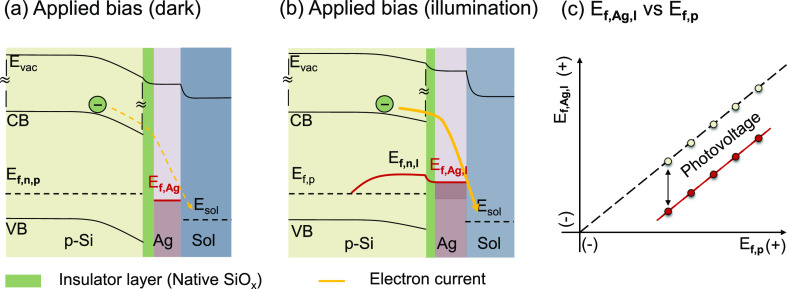
Fermi level (dark) and quasi-Fermi level (illuminated) alignment: energy band diagram of b-p-Si–Ag (a) at an applied bias under dark conditions, and (b) at the same applied bias under illuminated conditions. (c) Prediction of the quasi-Fermi level of Ag, *E*_f,Ag,l_ on b-p-Si–Ag (red) as a function of the bulk Fermi level, *E*_f,p_ (yellow). The difference between *E*_f,p_ (dashed line) and *E*_f,Ag,l_ (solid line) at each bias, that is, *E*_f,p_ is the corresponding photovoltage generated at the b-p-Si–Ag junction.

A model of the quasi-Fermi level alignment in the b-p-Si–Ag photocathode is depicted in Figure 2. With an applied cathodic bias (Figure 2a), the Fermi level of Ag, *E*_f,Ag_, shifts cathodically with the bulk Fermi level of p-Si, *E*_f,n,p_, with respect to the electrochemical potential of the solution, *E*_sol_. At the Schottky junction between p-Si and Ag, the band edges and the vacuum energy level (*E*_vac_) of p-Si shift with its *E*_f,n,p_ to the same extent (i.e., with constant band bending inside the Si space charge region). The electric potential (indicated by *E*_vac_) difference between the Si surface and that of the bulk solution is distributed among the potential drops at the Si/SiO_*x*_, SiO_*x*_/Ag, and the Ag/electrolyte junctions. Due to the potential drops at the former two junctions, the *E*_f,Ag_ is less negative than *E*_f,n,p_. Upon illumination (Figure 2b), the increase in the minority carrier (electron) density results in an upward shift of the quasi-Fermi level, *E*_f,n,l_. Accordingly, the quasi-Fermi level of Ag, *E*_f,Ag,l_ should also shift, along with an increase in the catalytic photocurrent. The difference between *E*_f,Ag,l_ and *E*_f,n,l_ depends on the charge transfer resistance at the Si/SiO_*x*_/Ag interfaces. The resulting difference between *E*_f,Ag,l_ and the bulk Fermi level of Si, *E*_f,p_ (i.e., the majority carrier Fermi level of p-type Si) is the photovoltage of interest (Figure 2c).

The shift in the Fermi level of Ag (*E*_f,Ag_, *E*_f,Ag,l_) is controlled by the change of the electrostatic potential it experiences, which can be measured by the vibrational Stark effect of adsorbed probe molecules. The electrostatic potential amplitude at the metal/adsorbed molecule monolayer/electrolyte interface undergoes linear decay within the dielectric self-assembled monolayer (SAM) layer and exhibits an exponential decrease toward the bulk solution, in accordance with the Gouy–Chapman theory, as described by Smith and White’s model.^25,27,28^ The corresponding electric field, proportional to the electric potential gradient, also decays from the electrode surface to the bulk solution. For adsorbed molecules on the Ag particle, their vibrational frequency changes linearly with the electric field through the vibrational Stark effect. Because the field strength changes linearly with the potential difference between the Ag particle and bulk solution, a shift in *E*_f_ of the Ag particle, caused by the shift of its electrostatic potential, should change the vibrational frequency of the adsorbed probe molecule. This gives rise to a linear dependence of the frequency on *E*_f_ of the Ag particle. This linear dependence can be measured directly with the same Stark effect probe molecules adsorbed on roughened Ag electrodes, for which the Fermi level is directly controlled by the potentiostat. Thus, the measured linear dependence can be used as a calibration curve to determine the (quasi-) Fermi level of the same metal on the oxide-covered semiconductor surface in contact with an electrolyte of the same composition.

**Figure 3:**
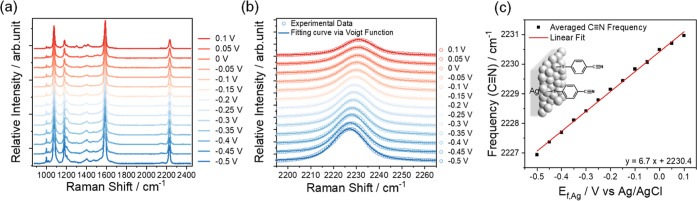
Stark effect calibration curve on roughened Ag electrodes. (a) Representative potential-dependent SERS spectra of 4-MBN on a bare silver electrode (Raman intensity: 16 × 10^5^ mW cm^–2^; solution: 0.2 M KHCO_3_). (b) Expanded view of the potential-dependent SERS spectra of the nitrile stretch region (open circles) and their fit (solid lines) to a Voigt lineshape function. (c) Measured averaged C≡N stretching frequency (filled squares) as a function of the Femi-level of the Ag electrode, *E*_f,Ag_ and a linear fit to the data according to the Stark effect-induced peak shift (solid line). The C≡N stretching frequency is averaged from those obtained under varying Raman intensity from 6 × 10^5^ to 16 × 10^5^ mW cm^–2^ (Figure S4). Inset: schematic representation of 4-MBN bound to a roughened silver electrode.

We first measured the C≡N stretching frequency of 4-MBN vs the Fermi level, that is, the calibration curve, on a roughened Ag electrode using a home-built SERS setup. The details of the setup are described in Section 3 of the Supporting Information. To obtain a SERS-active substrate, the Ag electrode was roughened via a cyclic oxidation–reduction treatment.^29^ The roughened Ag electrode was soaked in 10 mM acetonitrile solution of 4-MBN overnight to form the 4-MBN SAM. The in situ SERS measurements of the 4-MBN modified Ag electrode (Ag–4-MBN electrode) were conducted at applied potentials from −0.5 to 0.1 V with a potential interval of 0.05 V. At each potential, the SERS spectrum was collected under six Raman intensities varying from 6 × 10^5^ to 16 × 10^5^ mW cm^–2^. The SERS spectrum under each condition was collected with a detection time of 45 s. A representative set of Raman spectra measured at a Raman intensity of 16 × 10^5^ mW cm^–2^ are shown in Figure 3a, in which the spectra at different potentials have been displaced vertically. These spectra show four characteristic peaks at 1077.9, 1177.8, 1585.6, and 2226.9 cm^–1^ (at −0.5 V), in agreement with previous reports.^28,30,31^ The peaks at 1585.6 and 2226.9 cm^–1^ are assigned to the C=C ring stretching and C≡N stretching modes, respectively.^28,30,31^ The peaks at 1077.9 and 1177.8 cm^–1^ have been assigned to C–S stretching, and the aromatic C–H in-plane stretching, respectively,^30,31^ although different assignments have also been reported.^32,33^ We have limited this study to the potential range of −0.5 to 0.1 V because the thiol group starts to desorb from the Ag surface at applied potentials more negative than ∼−0.8 V (see Supporting Information, Section 4.3) and the oxidation of the Ag surface occurs at applied potentials more positive than +0.1 V.^34^ Within this potential range, the spectra are reproducible and independent of Raman laser intensity, as shown in Figure S4.

All four characteristic peaks of 4-MBN experience frequency shifts to different extents (Figure S3), consistent with previous reports.^30^ For this work, we will focus on the C≡N stretching mode because it shows the largest shift and its Stark effect has been extensively examined.^30,34−36^ The expanded view of the data in the C≡N stretching mode region is shown in Figure 3b (open circles). These spectra can be fit to a Voigt lineshape function that contains a baseline offset, peak center frequency, peak area, Gaussian, and Lorentzian full width at half-maximum as the five fitting parameters (see Supporting Information, Section 4). The fitted spectra are shown in Figure 3b (solid line), and fitting parameters are listed in Table S1. The C≡N stretching frequency obtained from the fit is plotted as a function of the Ag Fermi level in Figure S4, along with results measured under Raman light intensities varying from 6 × 10^5^ to 14 × 10^5^ mW cm^–2^, showing good agreement among measurements of different Raman laser intensities. These results are averaged to produce the plot of C≡N frequency versus Ag Fermi level shown in Figure 3c. As the Ag Fermi level is increased from −0.5 to 0.1 V (vs Ag/AgCl), the frequency of the C≡N stretching mode shifts from 2226.9 ± 0.02 to 2231.0 ± 0.02 cm^–1^. The dependence of the C≡N stretching frequency on the Fermi level of Ag can be well fit to a linear function: *v*_C≡__N_ = [ 6.7 × *E*_f,Ag_ (V, vs Ag/AgCl) + 2230.4] cm^–1^. This relationship will be used as a calibration curve to determine the quasi-Fermi level of Ag nanoparticles on the b-p-Si–Ag photocathode under illumination.

**Figure 4:**
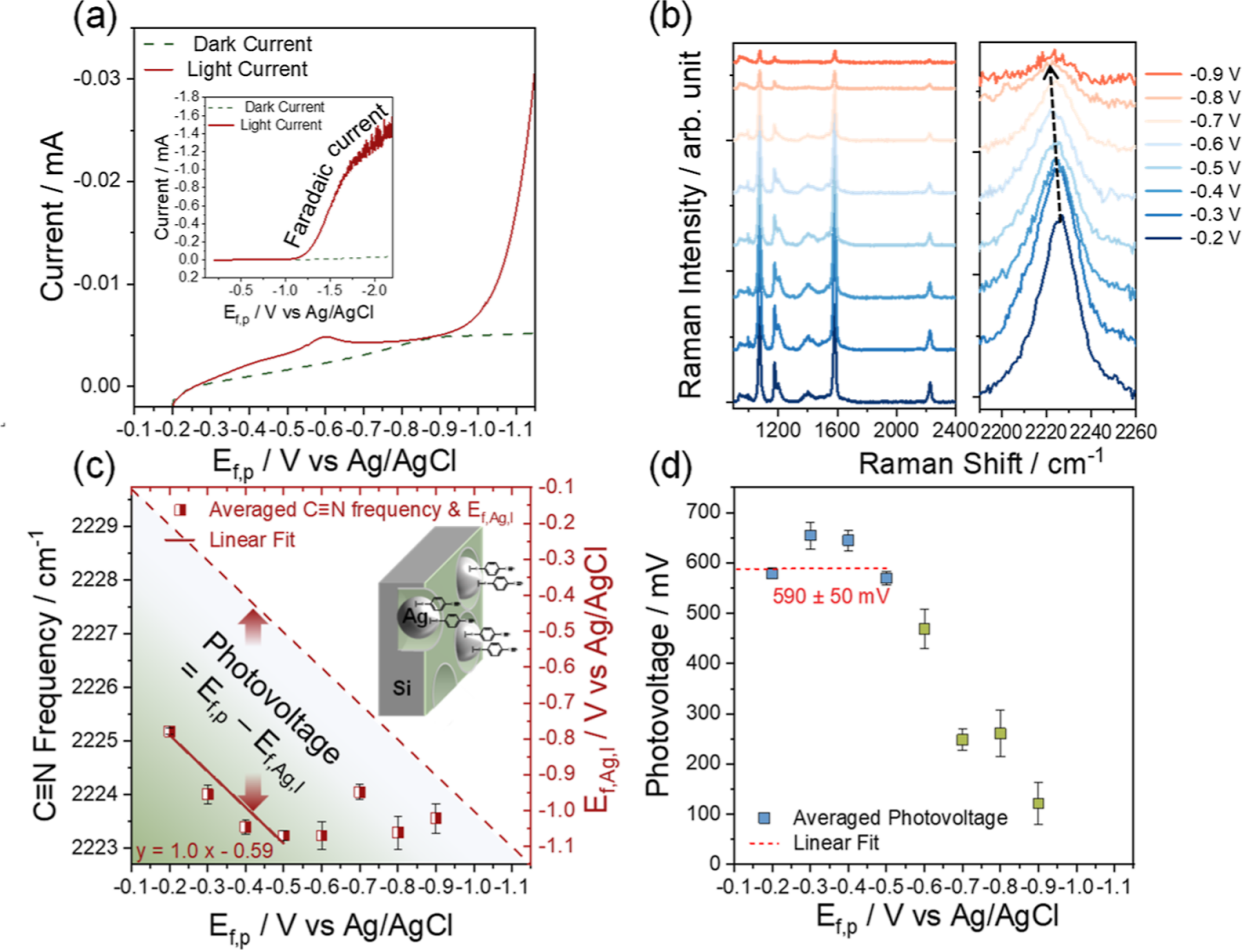
(a) *J*–*V* curve of the b-Si–Ag–4-MBN photocathode (illumination conditions: 6 × 10^5^ mW cm^–2^ Raman laser) inset: *J*–*V* curve with larger potential window from −0.2 to −2.2 V. (b) Potential-dependent SERS spectra (left) and zoomed-in spectra of the C≡N stretching peaks (right) under applied potentials from −0.2 to −0.9 V. (c) Averaged C≡N stretching frequency of 4-MBN and calibrated average quasi-Fermi level of Ag, *E*_f,Ag,l_, on b-p-Si–Ag from five successive measurements at each majority carrier Fermi level in p-type Si, *E*_f,p_, and (d) averaged photovoltage, that is, *E*_f,p_ – *E*_f,Ag,l_, as a function of *E*_f,p_. Data points from −0.6 to −0.9 V in green cannot represent the real photovoltage due to the desorption of 4-MBN.

Studies of b-p-Si–Ag–4-MBN photocathodes were carried out under 6 × 10^5^ mW cm^–2^ Raman laser illumination. A representative photocurrent measurement result is shown in Figure 4a, which reveals the appearance of a reductive desorption peak of 4-MBN with an onset potential of ∼−0.5 V. Similar reductive desorption peaks have been reported previously in thiol-anchored molecules on metal electrodes.^37,38^ The inset in Figure 4a shows an increasing cathodic current at more negative applied potentials and a photocurrent onset potential of ∼−1.0 V. The SERS spectra of the photocathode were collected in situ under a cathodic scan within the applied potential (*E*_f,p_) range from −0.2 to −0.9 V with a potential interval of 0.1 V. At each potential, five successive measurements of SERS spectra were recorded using a detection time of 60 s. At more negative potentials, the observed signal becomes too small to allow reliable SERS measurement due to the desorption of the 4-MBN molecule. Similar desorption phenomena were also observed for 4-MBN molecules on roughened Ag electrodes in the dark (Figure S6). The representative SERS spectra of b-p-Si–Ag–4-MBN photocathodes in the cathodic scan are shown in Figure 4b (left). The spectra show four characteristic peaks at 1075.0, 1175.9, 1582.7, and 2225.4 cm^–1^ (at −0.2 V) and similar bias-dependent shifts, consistent with those observed with the Ag–4-MBN electrodes. Similarly, our analysis focuses on the Stark shift of the C≡N stretching mode.

Shown in Figure 4b (right) is the expanded view of the spectra in the C≡N stretching mode region. From −0.2 to −0.5 V, the C≡N stretching frequency shifts toward a lower wavenumber, but from −0.6 V to −0.9 V, the peak position deviates from the red shift observed at less negative potentials. These spectra are fitted to a Voigt lineshape function following the same procedure as the result in Figure 3, and the fitting results are shown in Figure S5 and Table S2. The C≡N stretching peak frequencies obtained from five successive measurements at each potential are averaged to illustrate the potential-dependent Stark shift of the C≡N stretching peak (Figure 4c). The spectral features of 4-MBN on the b-Si–Ag photocathode differ from those of Ag electrodes in two significant ways. The frequencies of the C≡N stretching mode on the b-Si–Ag photocathode (Figure 4c) is red-shifted from those on roughened Ag electrodes at the same external bias applied on Si (*E*_f,p_) and Ag (*E*_f,Ag_), respectively. Furthermore, 4-MBN molecules on the b-Si–Ag photocathode desorb at less cathodic applied potentials compared to the same molecules on roughened Ag electrodes. As shown in Figure S6, the integrated Raman intensity of the C≡N stretching mode of 4-MBN molecules on roughened Ag electrodes starts to decrease when the applied potential is more negative than ∼−0.8 V (vs Ag/AgCl), while for b-Si–Ag electrodes, the Raman intensity of the same adsorbates starts to decrease at −0.2 V (Figure S7). Both observations suggest that the quasi-Fermi level on the b-Si–Ag photocathode is significantly more negative than the potential applied to the Si electrode, leading to the more anodic onset of adsorbate desorption and the lower C≡N stretching mode frequency.

The quasi-Fermi level on Ag nanoparticles of the b-Si–Ag photocathode can be determined by using the calibration equation that relates the C≡N frequency to the Ag Fermi level obtained from the bare-Ag–4-MBN measurement discussed above (Figure 3c). Each measured average C≡N stretching frequency for b-Si–Ag–4-MBN corresponds to an average quasi-Fermi level of Ag, *E*_f,Ag,l_. The *E*_f,Ag,l_ values obtained in this way are plotted as a function of the applied potential on Si, that is, the majority carrier Fermi level of Si, *E*_f,p_ in Figure 4c. The diagonal line in Figure 4c represents a situation of zero photovoltage, that is, *E*_f,p_ = *E*_f,Ag,l_. All data points are below this diagonal line, indicating that *E*_f,Ag,l_ is more negative than *E*_f,p_ for the b-Si–Ag photocathode. The average C≡N stretching frequency decreased linearly from ∼2225.2 ± 0.06 to 2223.2 ± 0.09 cm^–1^ under cathodic applied potentials (*E*_f,p_) from −0.2 to −0.5 V (Figure 4c), which corresponds to a shift in *E*_f,Ag,l_ from ∼−0.78 ± 0.01 to −1.07 ± 0.01 V (Figure 4c). Within this potential range, the relationship between *E*_f,Ag,l_ and *E*_f,p_ can be fitted to a linear relationship of *E*_f,Ag,l_ = *E*_f,p_ – 0.59. Our result suggests that on average, *E*_f,Ag,l_ is 0.59 ± 0.05 V more negative than *E*_f,p_, which corresponds to the photovoltage at the Si|SiO_*x*_|Ag junction (Figure 4d). At more cathodic potentials, the C≡N frequency appears to level off. Similar phenomena have been reported previously, although the exact origin is not understood. This has been attributed to a change of potential drops when there is an increase in current.^25,39^ It could also be caused by the change in the interaction between the electrode and the adsorbate near the desorption potential.^40^ For this reason, we limit our analysis of the photovoltage to the potential region of −0.2 to −0.5 V.

## Discussion

The photovoltage of silicon-based semiconducting photoelectrodes has been theoretically predicted to be less than 720 mV, limited by the Auger process.^41−43^ Several studies have reported photovoltages of approx. 500–620 mV at Si-based photoelectrodes with various configurations.^7,44−46^ Thus, the photovoltage reported here for the b-Si–Ag photocathode is consistent with previous reports. The photovoltage value can also be measured as the potential difference at the same current density between the photocurrent of a p-type b-Si–Ag photocathode and the dark current of an n-type b-Si–Ag cathode, which is ∼300 mV, significantly smaller than that measured by the spectroscopic method reported here.^23^ It should be noted that the photocurrent-based method reports the average photovoltage of the whole electrode, averaging over sites with and without Ag nanoparticles in the b-Si–Ag photoelectrode. The spectroscopic method described in this work probes the photovoltage exclusively at the Ag NPs-covered sites through the selective adsorption of 4-MBN molecules on Ag. Our method assumes that the electrostatic profiles of the double layer are identical at the Ag/electrolyte and Si/Ag/electrolyte interfaces when the Ag Fermi levels are the same. Any deviation from this assumption would give rise to an error in the measured photovoltage.

## Conclusions

In summary, we have developed an in situ, contactless spectroscopic method to directly probe the quasi-Fermi level of the metal layer in MIS photoelectrodes. By attaching a SERS reporter, the 4-MBN molecule, to a bare Ag electrode, a calibration relationship between the C≡N stretching frequency and the Fermi level of Ag was obtained. The quasi-Fermi level of Ag at a CO_2_ reduction photoelectrode, b-p-Si–Ag, at different applied potentials could then be determined from the potential-dependent Stark shift of the C≡N stretch via the calibration relation. The photovoltage at the Si/SiO_*x*_/Ag junction was measured to be ∼590 mV. We anticipate that this vibrational Stark probe method can be widely employed to probe the Fermi level (dark) and quasi-Fermi level (light) of various photoelectrodes, as long as an appropriate SERS reporter molecule can be selectively attached to the metal layer of interest and a calibration relation can be obtained. By directly quantifying the potential drop at the insulator layer and assessing the photovoltage of heterogeneous MIS junctions, this approach should be a valuable tool in the design of MIS electrodes with enhanced photovoltages.

## Supporting Information Available

Additional experimental and data analysis details, photoelectrochemistry and spectroscopy setup, and details on the spectra fitting (PDF)
