# Supplementary material for: Direct Vibrational Stark Shift Probe of Quasi-Fermi Level Alignment in Metal Nanoparticle Catalyst-Based Metal–Insulator–Semiconductor Junction Photoelectrodes
Source: J Am Chem Soc. 2023 Jun 22;145(26):14260–6. doi: 10.1021/jacs.3c02333 (PMC10326872; doi:10.1021/jacs.3c02333)
Supplement: Supplementary file 1 — ja3c02333_si_001.pdf [file ja3c02333_si_001.pdf]

Direct Vibrational Stark Shift Probe of Quasi-Fermi Level Alignment in Metal Nanoparticle Catalyst-Based Metal-Insulator-Semiconductor Junction Photoelectrodes

Sa Suo[1], Colton Sheehan[2], Fengyi Zhao[1], Langqiu Xiao[2], Zihao Xu[1], Jinhui Meng[1], Thomas E. Mallouk[2*], Tianquan Lian[1*]

[1]Department of Chemistry, Emory University, Atlanta, Georgia 30322, United States

[2]Department of Chemistry, University of Pennsylvania, Philadelphia, Pennsylvania 19104, United States

Table of Contents

1. Material Preparation 1

1.1 Ag electrode preparation 1

1.2 b-Si-Ag photoelectrode preparation 2

1.3 SAM layer preparation 2

2. (Photo)electrochemical Setup 2

3. Surface-enhanced Raman spectroscopy (SERS) Measurements 3

4. SERS spectra and fitting 3

4.1 Ag-4-MBN electrode 4

4.2 b-Si-Ag-4-MBN photoelectrode 6

4.3 Desorption of 4-MBN from Ag and b-Si-Ag electrodes 7

1. Material Preparation

1.1 Ag electrode preparation

A 0.5mm-thick, 0.5 cm × 1 cm Ag foil (thickness 0.5 mm, 99.99% trace metals basis) was used to fabricate bare Ag electrodes. To obtain the surface-enhanced Raman signal of 4-MBN on the Ag electrode, the Ag electrode was first mechanically polished and then roughened via an oxidation-reduction cycle (ORC) treatment.[1] The ORC treatment was carried out by using a three-electrode setup, which is described in section 2. The Ag electrode was emerged in 0.1 M KCl solution, and first oxidized with an applied potential ramp from -0.15 V to 0.65 V at a scan rate of 0.01 V s^{-1}. Then, reduction of the Ag electrode was done by ramping the potential from -1.25 V to -1.45 V at a scan rate of 0.001 Vs^{-1}. The color of the Ag electrode surface changed from metallic

silver to black (during the oxidation) and then to white (during the reduction). Both the Ag foil and KCl were purchased from Sigma-Aldrich.

1.2 b-Si-Ag photoelectrode preparation

Electrodes were prepared using single side polished, low doped p-type (1-10 Ω·cm) and degenerately doped n-type silicon wafers purchased from University Wafer. Wafers were cut into 1 cm × 1 cm pieces and sonicated in a mixture of acetone and water. The back side was rigorously scratched, and pieces were soaked in 5 wt% aqueous HF solution for 90 seconds. Insulated copper wires were contacted using GaIn eutectic (ThermoScientfic) and colloidal silver paste (Delta Technologies). Back contacts were covered in a thick film of Loctite EA 9460 Epoxy and allowed to cure for at least one day.

b-Si-Ag electrodes were fabricated via a metal assisted etching technique previously described.[2] Wired electrodes were initially soaked in a 5 wt% HF solution for 90 seconds. The electrodes were then transferred to a 1 mM aqueous solution of $AgNO_3$ in 1 wt% HF and soaked for 1 minute to electrolessly deposit silver particles. The electrodes were then soaked for 15 minutes in a 1 wt% H_2O_2, 1 wt% HF solution to be made porous, and silver particles were then removed with a 10-minute soak in 35 wt% HNO_3. Silver particles were then redeposited on the surface by repeating the initial 5 wt% HF and 1 mM $AgNO_3$ in 1 wt% HF steps. Between each step, electrodes were rigorously rinsed with water and blown dry with inert gas. HF (47.5 wt%), $AgNO_3$, H_2O_2 (30 wt%), and HNO_3 (70 wt%) were purchased from Spectrum Chemical, Sigma-Aldrich, Thermo Fisher Scientific and EMD Millipore, respectively.

1.3 SAM layer preparation

Both the roughened Ag electrode and the b-Si-Ag electrode were soaked in a 10 mM acetonitrile solution of 4-MBN overnight at room temperature to form the 4-MBN self-assembled monolayer (SAM).[3] Both Ag electrode and b-Si-Ag electrode were sonicated in acetonitrile solution for 1 minute prior to the Raman measurement. 4-MBN was purchased from Biosynth.

2. (Photo)electrochemical Setup

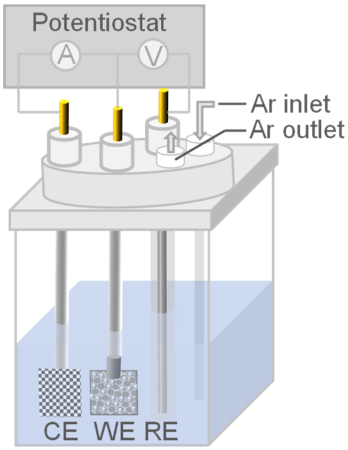

Figure S1. Scheme of the three-electrode (photo)electrochemical setup. (WE: working electrode; CE: counter electrode; RE: reference electrode)

The solution for the (photo)electrochemical measurement is 0.2M $KHCO_3$ (pH = 8.2). The working electrode (WE) is b-p-Si-Ag NPs-4-MBN or roughened Ag electrode. The reference electrode (RE) is Ag/AgCl/1M KCl (Gaoss Union). The counter electrode (CE) is Pt mesh (Gaoss Union). $KHCO_3$ (BioUltra, ⩾99.5%) was purchased from Sigma-Aldrich.

3. Surface-enhanced Raman spectroscopy (SERS) Measurements

The SERS measurements were conducted via a home-built Raman setup. The Raman laser source was HeNe 632.8nm (Thorlabs HNL210LB). The laser light was directed to the electrode surface, passing through an neutral density filter for tuning the fluence intensity and focused by a 10X microscope objective (10X Olympus Plan Achromat Objective, 0.25 NA, 10.6 mm WD, from Thorlabs, RMS10X) at the sample surface. The scattered light from the sample spot was collected by the 10X microscope objective. The collected Raman signal was filtered by a 633 nm single-notch filter (StopLine, Semrock), directed to be dispersed by a spectrograph (Shamrock, Andor) and then detected by an electron-multiplied charge coupled device (EMCCD, Newton, Andor).

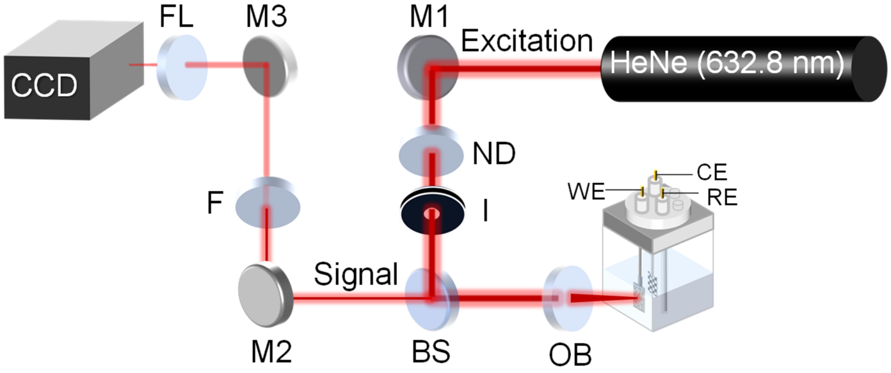

Figure S2. Scheme of the *in-situ* surface-enhanced Raman spectroscopy (SERS) setup. Light source: 632.8 nm Raman laser. M: mirror; ND: neutral density filter; BS: beam splitter; OB: 10X objective lens; F: 633 nm filter; FL: focus lens.

4. SERS spectra and fitting

The Voigt function is used to fit the C≡N stretching frequency on both Ag-4-MBN and b-Si-Ag-4-MBN electrodes:

$$y = y_0 + \int_{-\infty}^{\infty} \frac{2A}{\pi} \frac{W_L}{4(x-x_c)^2 + W_L^2} \sqrt{\frac{4ln2}{\pi}} \frac{e^{-\frac{4ln2}{W_G^2}x^2}}{W_G} dx$$

, where y_0 is the baseline offset, x_c is the center wavenumber of the peak, A is the area of the peak, W_G and W_L are the Gaussian and Lorentzian full width at half maximum (FWHM), respectively.

4.1 Ag-4-MBN electrode

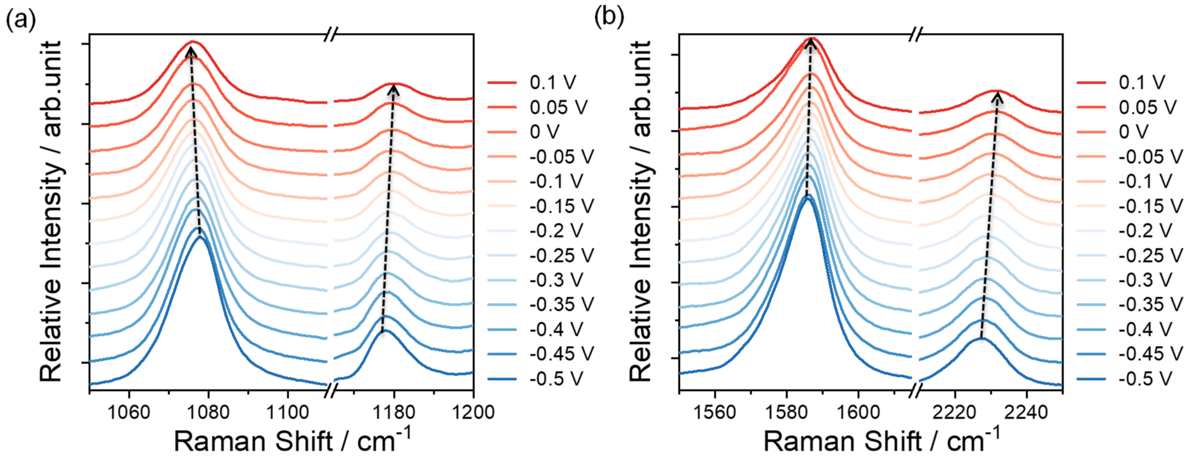

Figure S3. Zoomed-in spectra of Figure 3a: potential-dependent SERS spectra of the Ag-4-MBN under anodic scan from -0.5 V (dark blue) to 0.1 V (dark red) for (a) the C-S stretching and the aromatic C-H in-plane stretching, and (b) the C=C ring stretching and C≡N stretching modes, from left to right. (Illumination conditions: 16×10^5 mW/cm^2 Raman laser; Solution: 0.2 M $KHCO_3$)

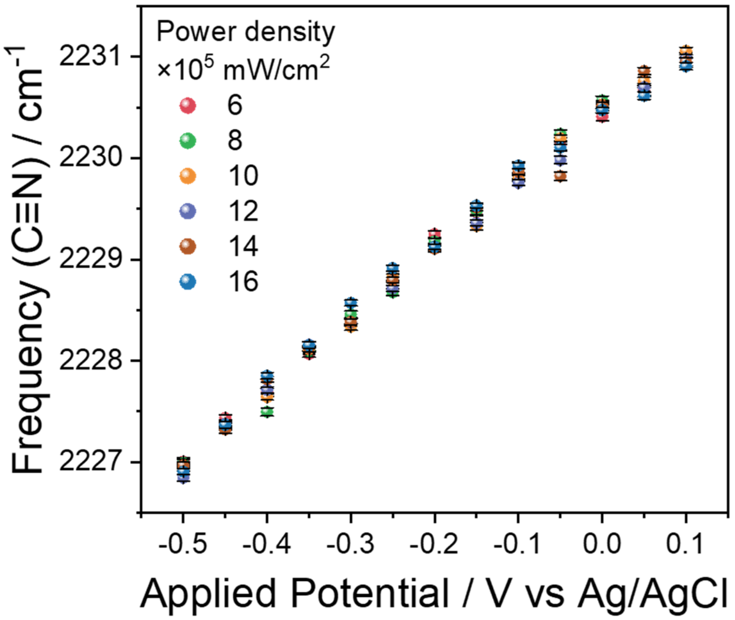

Figure S4. C≡N stretching frequencies as a function of applied potential, *i.e.*, Ag Fermi-level, under varying Raman intensities from 6×10^5 to 16×10^5 mW/cm^2. The SERS spectra are measured under an anodic scan from -0.5 V to 0.1 V. The Raman power-dependent SERS spectra are collected at each potential with light intensities ranging from 6×10^5 to 16×10^5 mW/cm^2.

Table S1. Representative summary of the fitting parameters for the C≡N stretching modes in Ag-4-MBN electrodes (Illumination conditions: 16×10^5 mW/cm^2 Raman laser)

Potential / V	y_0	x_c	A	w_G	w_L
-0.5	-161.5 ± 19.3	2226.9 ± 0.03	292907.8 ± 2875.8	11.4 ± 0.3	8.9 ± 0.4
-0.45	-126.6 ± 18.2	2227.4 ± 0.03	259969.5 ± 2685.6	11.8 ± 0.3	8.1 ± 0.4
-0.4	-134 ± 17.4	2227.9 ± 0.03	260446.3 ± 2567.6	10.9 ± 0.3	8.9 ± 0.4
-0.35	-149.2 ± 15.8	2228.2 ± 0.03	248110.3 ± 2340.2	10.1 ± 0.3	10 ± 0.3
-0.3	-138.1 ± 14	2228.6 ± 0.03	230528.7 ± 2058.5	10.1 ± 0.3	9.5 ± 0.3
-0.25	-145.9 ± 13.1	2228.9 ± 0.03	229715.4 ± 1942.7	10.1 ± 0.3	10 ± 0.3
-0.2	-143.6 ± 11.2	2229.1 ± 0.03	219827.3 ± 1680.4	10.3 ± 0.3	10.1 ± 0.3
-0.15	-126.8 ± 12	2229.5 ± 0.03	216187.8 ± 1807.3	10.1 ± 0.3	10.6 ± 0.3
-0.1	-115.6 ± 12.3	2229.9 ± 0.03	191491 ± 1843.9	11.2 ± 0.3	9.5 ± 0.4
-0.05	-129.6 ± 11.9	2230.1 ± 0.03	193430.9 ± 1808	9.6 ± 0.4	11.3 ± 0.3
0	-104.4 ± 9.3	2230.5 ± 0.03	173658.3 ± 1410.6	9.4 ± 0.3	11.4 ± 0.3
0.05	-121.9 ± 11.7	2230.6 ± 0.04	174359 ± 1819.8	10.3 ± 0.4	11.8 ± 0.4
0.1	-80.6 ± 10.4	2230.9 ± 0.04	141660.8 ± 1533.6	9.9 ± 0.4	9.9 ± 0.4

4.2 b-Si-Ag-4-MBN photoelectrode

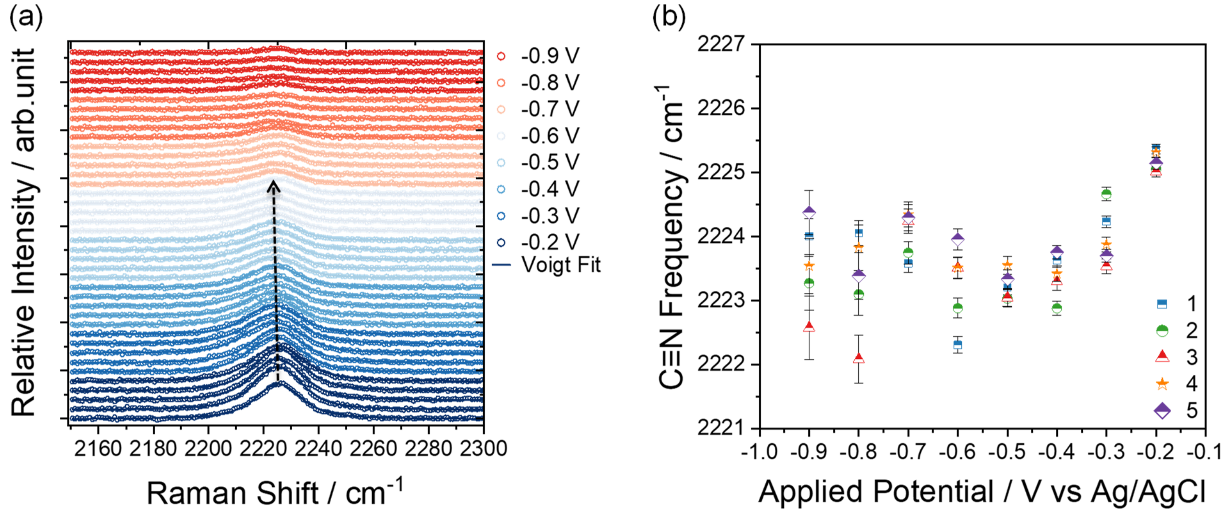

Figure S5. (a) Potential-dependent zoomed-in SERS spectra of the C≡N stretching peaks of b-Si-Ag-4-MBN under applied potentials, *i.e.*, majority carrier Fermi-level of p-Si, from -0.2 V to -0.9 V with 5 successive measurements at each potential (vertically stacked from bottom to top). The circle markers represent the original spectra data, and the solid line shows the fitted data by applying the Voigt function. (Illumination conditions: 6×10^5 mW/cm^2 Raman laser; Solution: 0.2M $KHCO_3$) (b) Stark shift of the C≡N stretching frequency as a function of the applied potential. The 5 successive measurements under each potential are represented by different markers, noted as No. 1-5.

Table S2. Summary of the fitting parameters for the C≡N stretching modes in b-Si-Ag-4-MBN electrodes.

Potential / V	No.	y_0	x_c	A	w_G	w_L
-0.2	1	-29.2 ± 5.1	2225.4 ± 0.1	41712.2 ± 856.7	10.5 ± 0.9	14.5 ± 0.9
	2	-36 ± 6.2	2225.1 ± 0.1	51293.6 ± 1037	13.1 ± 0.8	12.6 ± 0.9
	3	-26.7 ± 6.7	2225 ± 0.1	50134.2 ± 1144.8	14.4 ± 0.9	12 ± 1.0
	4	-37.1 ± 6.8	2225.3 ± 0.1	52109.8 ± 1167	12.2 ± 1	14.1 ± 1.0
	5	-27.7 ± 6.9	2225.1 ± 0.1	46280.6 ± 1219.6	13.8 ± 1.1	14.2 ± 1.2
-0.3	1	-21 ± 6.5	2224.2 ± 0.1	42957.3 ± 1127	16.5 ± 1	11.1 ± 1.2
	2	-43.4 ± 7.2	2224.7 ± 0.1	42964.7 ± 1246	13.3 ± 1.3	14.2 ± 1.3
	3	-17.9 ± 7.7	2223.5 ± 0.1	41060.6 ± 1398.1	18.6 ± 1.3	11.2 ± 1.7
	4	-31.5 ± 7.3	2223.9 ± 0.1	41096.3 ± 1317.5	14.4 ± 1.4	15.2 ± 1.6
	5	-19.1 ± 6.2	2223.7 ± 0.1	35824.3 ± 1079.5	16.7 ± 1.1	10.7 ± 1.4
-0.4	1	-19.2 ± 5.8	2223.6 ± 0.1	34384.2 ± 1019.5	12.7 ± 1.3	15 ± 1.4

	2	-10.6 ± 5.2	2222.9 ± 0.1	27491.7 ± 888.6	17.8 ± 1.2	9 ± 1.5
	3	-15.2 ± 6.1	2223.3 ± 0.1	27809.7 ± 1073.5	15.9 ± 1.6	12.5 ± 1.9
	4	-17 ± 5	2223.4 ± 0.1	26189.5 ± 864.1	14.4 ± 1.4	12.9 ± 1.5
	5	-19.4 ± 4.7	2223.8 ± 0.1	26084.8 ± 793.5	13.8 ± 1.2	12.5 ± 1.4
-0.5	1	-8.8 ± 4.7	2223.2 ± 0.1	24981.6 ± 810	16.3 ± 1.2	10.6 ± 1.5
	2	-9.2 ± 5.3	2223 ± 0.1	22916.7 ± 910.5	17.3 ± 1.4	9.5 ± 1.9
	3	-8 ± 5.3	2223 ± 0.1	22905.3 ± 913.4	18.1 ± 1.5	9.3 ± 1.9
	4	-20.2 ± 5.3	2223.6 ± 0.1	24434.6 ± 928.7	12.7 ± 1.7	14.7 ± 1.7
	5	-6.3 ± 5.4	2223.3 ± 0.1	21901.1 ± 910.9	15.5 ± 1.6	10.9 ± 1.9
-0.6	1	-5.6 ± 4.6	2222.3 ± 0.1	21190 ± 790	15.8 ± 1.4	11.4 ± 1.7
	2	-11.3 ± 4.6	2222.9 ± 0.2	17558.1 ± 777.6	15.7 ± 1.7	10.8 ± 2
	3	-2.2 ± 4.6	2223.5 ± 0.2	15541 ± 750.2	16.8 ± 1.6	7.6 ± 2.1
	4	-12.5 ± 4.6	2223.5 ± 0.2	15705 ± 766.4	14.2 ± 1.8	10.8 ± 2.1
	5	-6.2 ± 4.6	2224 ± 0.2	15256.1 ± 749.7	16.2 ± 1.6	7.7 ± 2.1
-0.7	1	-8.9 ± 3.8	2223.6 ± 0.1	15741.6 ± 631.8	12.6 ± 1.6	12.7 ± 1.7
	2	-12.9 ± 4	2223.7 ± 0.2	13363.3 ± 661.2	11.2 ± 2.1	13.6 ± 2.1
	3	-10.2 ± 4.6	2224.2 ± 0.2	14783.8 ± 769.4	10.3 ± 2.4	14.7 ± 2.2
	4	-14.4 ± 4.5	2224.4 ± 0.2	14856.6 ± 748.9	7.1 ± 3.2	17.2 ± 2.1
	5	-9.1 ± 4.6	2224.3 ± 0.2	13507.7 ± 764.9	10.1 ± 2.6	14.3 ± 2.3
-0.8	1	3.7 ± 3.8	2224.1 ± 0.2	10116.7 ± 598.6	15.9 ± 1.9	7.1 ± 2.5
	2	-4.6 ± 4.1	2223.1 ± 0.3	7158.2 ± 631.8	4.6 ± 6.4	15.6 ± 3.2
	3	-2.7 ± 4.1	2222.1 ± 0.4	6063.9 ± 665.3	12.8 ± 4.1	11.2 ± 4.6
	4	-5.2 ± 3.7	2223.8 ± 0.4	6482.5 ± 597.3	5.4 ± 6.8	17.2 ± 3.6
	5	2.7 ± 3.4	2223.4 ± 0.4	4209.8 ± 504.7	15.3 ± 3.3	4.4 ± 4.6
-0.9	1	-1.3 ± 3.9	2224 ± 0.3	6960.4 ± 617.7	17.4 ± 2.7	4.8 ± 3.8
	2	0.4 ± 3.5	2223.3 ± 0.4	4422.4 ± 547.7	12.1 ± 4.5	10.4 ± 4.9
	3	-0.1 ± 3.6	2222.6 ± 0.5	3744.3 ± 554.6	11.1 ± 5.2	10.1 ± 5.6
	4	2.6 ± 2.1	2223.5 ± 0.4	2615.9 ± 160.2	16.5 ± 1.1	0.0 ± 0.0
	5	36.3 ± 4.6	2171.6 ± 4.3	-1718.1 ± 559.9	35.2 ± 12	0.0 ± 0.0

4.3 Desorption of 4-MBN from Ag and b-Si-Ag electrodes

The potential-dependent C≡N stretching bands on Ag-4-MBN electrode were collected under a cathodic scan from 0.1 V to -1.35 V. The band intensity (illustrated as the band area) of the C≡N stretching modes first linearly increase with more cathodic potentials from 0.1 V till -0.8 V. The increasing trend results from the potential-dependent variation in the nitrile orientation with respect to the surface normal, which is due to the altered electron distribution and thus electric dipoles of the molecules.[4–7] At potentials more cathodic than -0.8 V, the C≡N stretching peak area shows a continuous decrease, indicating that the 4-MBN starts to desorb from the Ag electrode surface concomitant with a reduced surface density of the SAM layer. At potentials more cathodic

than -1 V, the magnitude of the band area drops below the initial magnitude at 0.1 V and approaches zero after ~ -1.35 V. (Figure S6)

As is illustrated in the main text, the quasi-Fermi level of Ag, $E_{f,Ag,l}$ on the b-Si-Ag-4-MBN shifts from ~ -0.8 V to ~ -1.1V under the cathodic scan from applied potential of -0.2 V to -0.9 V. The band area of the C≡N stretching mode shows a linear decrease with respect to the applied potential, which is in accord with the 4-MBN desorption trend at potentials more negative than -0.8 V observed in the Ag-4-MBN electrode. (Figure S7)

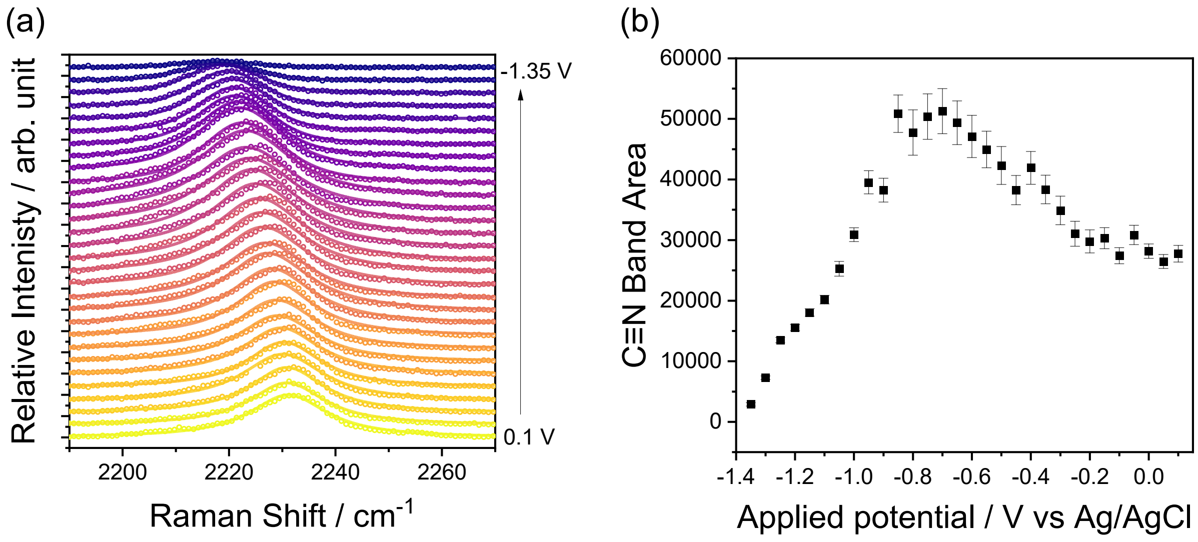

Figure S6. (a) SERS spectra of the C≡N stretching peaks of Ag-4-MBN under the cathodic scan from the applied potential, *i.e.*, Ag Fermi-level, of 0.1 V to -1.35 V. The circle markers represent the original spectrum data, and the solid line shows the fitted data via the Voigt function. (b) The SERS band area of C≡N stretching mode as a function of applied potential.

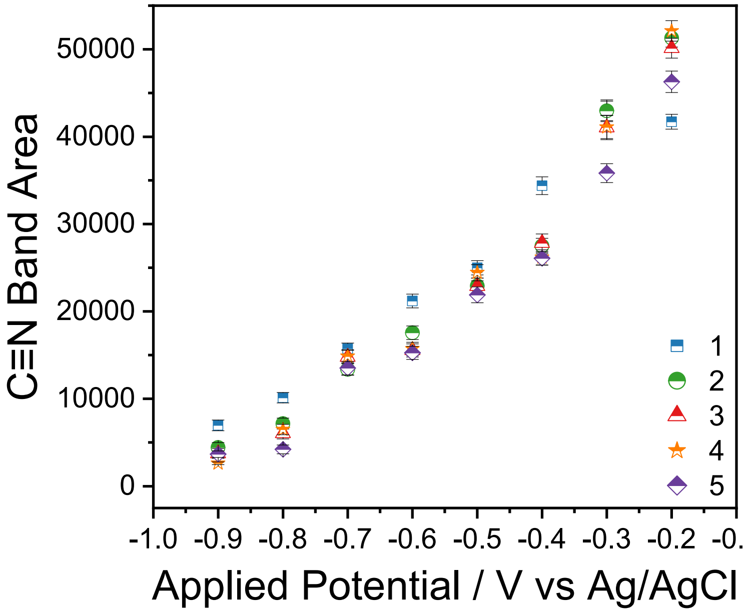

Figure S7. The SERS band area of C≡N stretching mode (in Fig. S4a) on b-Si-Ag-4-MBN photoelectrode as a function of applied potential, *i.e.*, majority carrier Fermi-level of p-Si.

(1) Huang, Y.-F.; Zhu, H.-P.; Liu, G.-K.; Wu, D.-Y.; Ren, B.; Tian, Z.-Q. When the Signal Is Not from the Original Molecule To Be Detected: Chemical Transformation of *Para* - Aminothiophenol on Ag during the SERS Measurement. *J. Am. Chem. Soc.* **2010**, *132* (27), 9244–9246.

(2) Kan, M.; Yan, Z. W.; Wang, X.; Hitt, J. L.; Xiao, L.; McNeill, J. M.; Wang, Y.; Zhao, Y.; Mallouk, T. E. 2-Aminobenzenethiol-Functionalized Silver-Decorated Nanoporous Silicon Photoelectrodes for Selective CO_2 Reduction. *Angew. Chem.* **2020**, *132* (28), 11559–11566.

(3) Pennathur, A. K.; Voegtle, M. J.; Menachekanian, S.; Dawlaty, J. M. Strong Propensity of Ionic Liquids in Their Aqueous Solutions for an Organic-Modified Metal Surface. *J. Phys. Chem. B* **2020**, *124* (34), 7500–7507.

(4) Delley, M. F.; Nichols, E. M.; Mayer, J. M. Interfacial Acid–Base Equilibria and Electric Fields Concurrently Probed by *In Situ* Surface-Enhanced Infrared Spectroscopy. *J. Am. Chem. Soc.* **2021**, *143* (28), 10778–10792.

(5) Staffa, J. K.; Lorenz, L.; Stolarski, M.; Murgida, D. H.; Zebger, I.; Utesch, T.; Kozuch, J.; Hildebrandt, P. Determination of the Local Electric Field at Au/SAM Interfaces Using the Vibrational Stark Effect. *J. Phys. Chem. C* **2017**, *121* (40), 22274–22285.

(6) Schkolnik, G.; Salewski, J.; Millo, D.; Zebger, I.; Franzen, S.; Hildebrandt, P. Vibrational Stark Effect of the Electric-Field Reporter 4-Mercaptobenzonitrile as a Tool for Investigating Electrostatics at Electrode/SAM/Solution Interfaces. *Int. J. Mol. Sci.* **2012**, *13* (6), 7466–7482.

(7) Di Martino, G.; Turek, V. A.; Lombardi, A.; Szabó, I.; de Nijs, B.; Kuhn, A.; Rosta, E.; Baumberg, J. J. Tracking Nanoelectrochemistry Using Individual Plasmonic Nanocavities. *Nano Lett.* **2017**, *17* (8), 4840–4845.
